# Angular Photochromic LC Composite Film for an Anti-Counterfeiting Label

**DOI:** 10.3390/polym10040453

**Published:** 2018-04-18

**Authors:** Yanzi Gao, Wenhuan Yao, Jian Sun, Kexuan Li, Lanying Zhang

**Affiliations:** 1 Department of Materials Science and Engineering, College of Engineering, Peking University, Beijing 100871, China; gaoyanzi@pku.edu.cn (Y.G.); sun6jian10@gmail.com (J.S.); 2 Key Laboratory of Polymer Chemistry and Physics of Ministry of Education, Peking University, Beijing 100871, China; 3 School of Chemistry and Chemical Engineering, Northwest Normal University, Lanzhou 620100, China; wenhuanyao@163.com; 4 Department of Applied Statistics and Science, Xijing University, Xi’an 710123, China

**Keywords:** liquid crystal, anti-fake, cholesteric phase, side-chain liquid crystal polymers

## Abstract

In the harsh application environment, improving the mechanical properties of liquid crystal materials is a fundamental and important problem in the design of anti-counterfeit materials. In this paper, by a stepwise polymerization of first, photo-polymerization and subsequently thermal-polymerization, a coexistent polymer dispersed network was first constructed in cholesteric liquid crystal materials containing a photo-polymerizable system of urethane acrylate and a thermo-polymerizable system of isocyanate. Results revealed that the coexistent polymer dispersed network exhibited largely enhanced mechanical performance, and the networks obtained by different methods had different contributions to the enhancement of the peel strength and toughness of the composite films. Then an angular photochromic anti-fake label based on a coexistent polymer dispersed network with enhanced mechanical and apparent angular discoloration characteristics, suitable for practical applications, was successfully achieved.

## 1. Introduction

Anti-fake technologies such as holograms [1], watermarks [2], coated labels [3], and so on have been developed to tackle counterfeiting problems for many decades. However, traditional anti-fake technologies are becoming known to counterfeiters and they are not able to meet the various demands from different fields. For the negotiable instrument field such as for currencies, bank documents, and so on, easy to be identified by the public is the preferred choice for anti-fake materials. Furthermore, due to the harsh application environment, good mechanical properties are also urgently needed to prevent materials from wearing out.

Liquid crystal (LC), which exhibits excellent optical, controllable, characteristic, and self-assembled soft matter properties, can be used as anti-fake material and has been widely reported previously [4,5,6]. Attributing to the unique helical supra-molecular structure, cholesteric LC (ChLC) could selectively reflect circularly polarized incident light whose handedness is identical with the helical axis [7]. The selective reflection phenomenon could be easily observed by the naked eye and also be detected by instruments. Moreover, the refection wavelength and circular polarization property of the reflected light could both be artificially tuned [8,9]. The reflection wavelength, λ = *nP*sinθ, where *n* = (*no* + *ne*)/2 is the average of the ordinary (*no*) and extraordinary (*ne*) refractive indices of the ChLC, *P* is the cholesteric pitch corresponding to the length of a 2π molecular rotation, and θ is the angle between the surface and viewing direction. Accordingly, for a specific ChLC material, the reflection colors (supposing that the reflection wavelength is in the visible region) is directly proportional to the viewing angle, which is very suitable for anti-fake purposes.

However, the mechanical properties of pure LC materials are not satisfactory for application. Reports have shown that there are many different methods to reinforce the mechanical properties of a material such as nanoparticle filling [10,11,12,13], mineral reinforcing [14,15,16], polymer composite [17,18,19], and so on. For research to improve the mechanical properties of the liquid crystal material, there is still a lack of attention. Based on the excellent mechanical properties and easy processing characteristics of polymers, Prof. H. Yang composited ethylene-vinyl acetate (EVA) with cholesteric side-chain liquid crystal polymers (ChSCLCP) to improve the mechanical property of infrared light shielding LC film [20]. By adjusting the ratios and manufacturing processes, the composite film was successfully prepared with mechanical properties as good as pure EVA film without loss of its transmittance. Another method to improve the mechanical properties was by introduction of a polymer network into LC materials. The polymer-dispersed liquid crystal (PDLC) system, which consists of a continuous polymer matrix with micro-sized LC droplets dispersed in it, can be manufactured either by UV light or thermal curing. During the polymerization, phase separation occurs and the LC forms a microphase separation structure in the polymer network. The strong interaction between the continuous polymer network and the substrate endows the PDLC film with strong peeling strength [21,22,23,24]. Prof. Yang invented a novel coexistent system of polymer-dispersed and polymer-stabilized liquid crystals (PD&SLCs), which forms a homeotropically aligned polymer network (HAPN) within the LC droplets after a microphase separation between the LC and the polymer matrix, and combines the advantages of both the PDLC and PSLC systems. Compared with the corresponding traditional PSLC film, a great improvement of shearing force was achieved in the as-made PD&SLC film [25,26]. Prof. Wang prepared a kind of light scattering display with body temperature controlled optical and thermal information storage properties based on a special “loofah-like gel network” of super strong liquid crystalline physical gel. The study found that the adding of gelators in host 5CB can relatively resist a great outer force [17].

In the present paper, a series of polymer dispersed cholesteric liquid crystalline film was prepared. Different from the work based on a single polymer dispersed network by photo-polymerization or thermal-polymerization previously reported, a coexistent polymer dispersed network by a stepwise polymerization of first photo-polymerization and subsequent thermal-polymerization was first realized; the impact of different polymer dispersed networks and ChLC materials on the mechanical and optical properties of the ChLC films was systematically investigated. Then an angular photochromic anti-fake label based on a coexistent polymer dispersed network with enhanced mechanical and apparent angular discoloration characteristics suitable for practical applications was successfully prepared.

## 2. Materials and Methods

### 2.1. Materials

The nematic LC SLC1717 (T_Cr-N_ <−40.0 °C, T_N-I_ = 91.8 °C) was purchased from Shijiazhuang Yongsheng Huatsing Liquid Crystal Co., Ltd. (Shijiazhuang, China). S811 (Jiangsu Hecheng Display Technology Co., Ltd., Nanjing, China) and urethane acrylate (UA, CN9178NS, Sartomer) were commercially available and used without any further purification. Poly-isophorone di-isocyanate (IPDI, 98%), tetra-ethylene glycol (TTEG, 99%), and other chemical reagents were used as received. ChSCLCP was obtained via conventional free radical polymerization of different liquid crystalline monomers—for details of the synthetic route refer to our previous work [27,28]—and the nematic LC monomer C6M was prepared in our own laboratory.

### 2.2. Measurements

A PerkinElmer DSC8000 (PerkinElmer, Waltham, MA, USA) with a mechanical refrigerator was used to obtain the phase transition of the polymers under dry nitrogen at a heating and cooling rate of 20 °C min^−1^; the temperature and heat flow scale were calibrated using zinc and indium as standards. Polarized optical microscopy (POM) was carried out on a Carl Zeiss Axio Vision SE64 polarized optical microscope (Carl Zeiss, Oberkochen, Germany) with a Linkam LTS420 hot stage. Spectral characterization was done by an unpolarized UV/Vis/IR spectrophotometer (Perkin–Elmer Lambda 950, PerkinElmer, Waltham, MA, USA) in transmission mode at normal incidence. The peeling strength experiment was practiced on a universal tensile test machine (Instron 5969, Instron, Boston, MA, United States) and the rate of extension was 0.5 mm s^−1^. The samples were sandwiched between two PET films to perform the peeling strength experiment from the horizontal direction of the film. The horizontal cross-section area was 1 cm^2^.

### 2.3. Preparation of the Samples

The prepared samples were mixed thoroughly in the specified proportions according to Table 1 until they were homogenized. Then, the mixture was filled into two layers of PET substrate, respectively, with a thickness of 20 ± 1 μm controlled by a spacer. After this, samples A1, A2, A3, and C1 were irradiated by a UV lamp (365 nm 35-W Hg lamp, PS135, UV Flood, Stockholm, Sweden) for 30 min at room temperature; samples B1, B2, B3, and C5 were thermally cured in an oven at 363.15 K for 7 h; sample C2, C3, C4, D1, D2, D3, and D4 were first irradiated by a UV lamp for 30 min, then thermal cured in an oven at 363.15 K for 7 h.

## 3. Results and Discussion

### 3.1. Mesomorphic and Optical Properties of the Cholesteric Liquid Crystal Materials

**Scheme 1:**
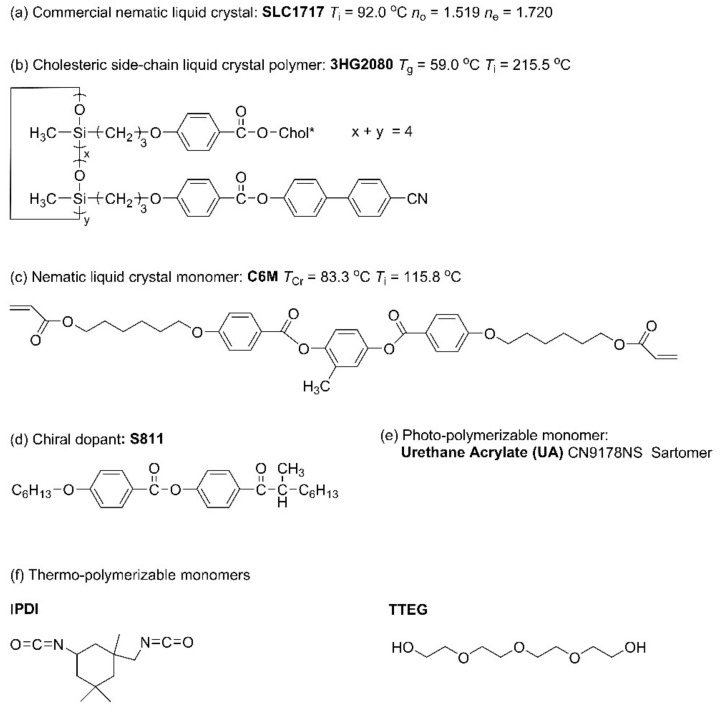
The chemical structures of the materials used.

Previous reports have shown that the center reflection wavelength of chiral compounds strongly relied on the content of the chiral component [29]. Accordingly, we designed three different ChLC systems, a small molecular weight liquid crystal system, a side-chain liquid crystal polymer system, and a polymerizable liquid crystal system, with selective reflective wavelength covering the visible range, as shown in Table 1. Scheme 1 exhibits the chemical structures and some basic physical parameters of the monomers, ChSCLCP, the small molecular weight nematic LC, etc. Among them, the small molecular weight nematic LC SLC1717 was a commercial product, the ChSCLCP was obtained via conventional free radical polymerization of different liquid crystalline monomers—for details of the synthetic route refer to our previous work [27,28]—and the nematic LC monomer C6M was prepared in our own laboratory. As expected, the three ChLC systems all exhibited a wide temperature range of the cholesteric phase, and the central selective reflection wavelengths were 525 nm, 668 nm, and 675 nm, respectively.

### 3.2. Dependence of the Polymer Dispersed Network on the Optical and Mechanical Properties

In our previous studies, we revealed that the introduction of a polymer dispersed network into LC could considerably improve the peeling strength of the material [25]. However, the polymer dispersed network can be obtained by either photo [21] or thermal [22] polymerization and the influence was still not clear of the preparation method of the polymer network on the mechanical properties and the interplay between them. As a consequence, we attempted to design a single polymer dispersed network by photo-polymerization or thermal-polymerization, as well as a coexistent polymer dispersed network by a step polymerization of photo- and thermal-polymerization, in order to find out the optimal resolution for mechanical performance improvement.

As shown in Table 2, a series of polymer dispersed ChLC films were prepared. For sample series A, which is denoted as A1–A3, a polymer dispersed network by photo-polymerization was introduced into the designed ChLCs, while in sample series B, denoted as B1–B3, a polymer dispersed network by thermo-polymerization was introduced. In sample series C, sample C1 was prepared by photo-polymerization, sample C5 was prepared by thermo-polymerization, samples C2, C3, and C4 were prepared by first photo-polymerization and then thermo-polymerization. For sample series D, denoted as D1 and D2, a polymer dispersed network by first photo-polymerization and then thermo-polymerization was introduced into the designed ChLCs. The optical and mechanical properties of all the samples were characterized by a combination of POM, UV/Vis/IR spectra, and peeling strength measurement. 

**Figure 1:**
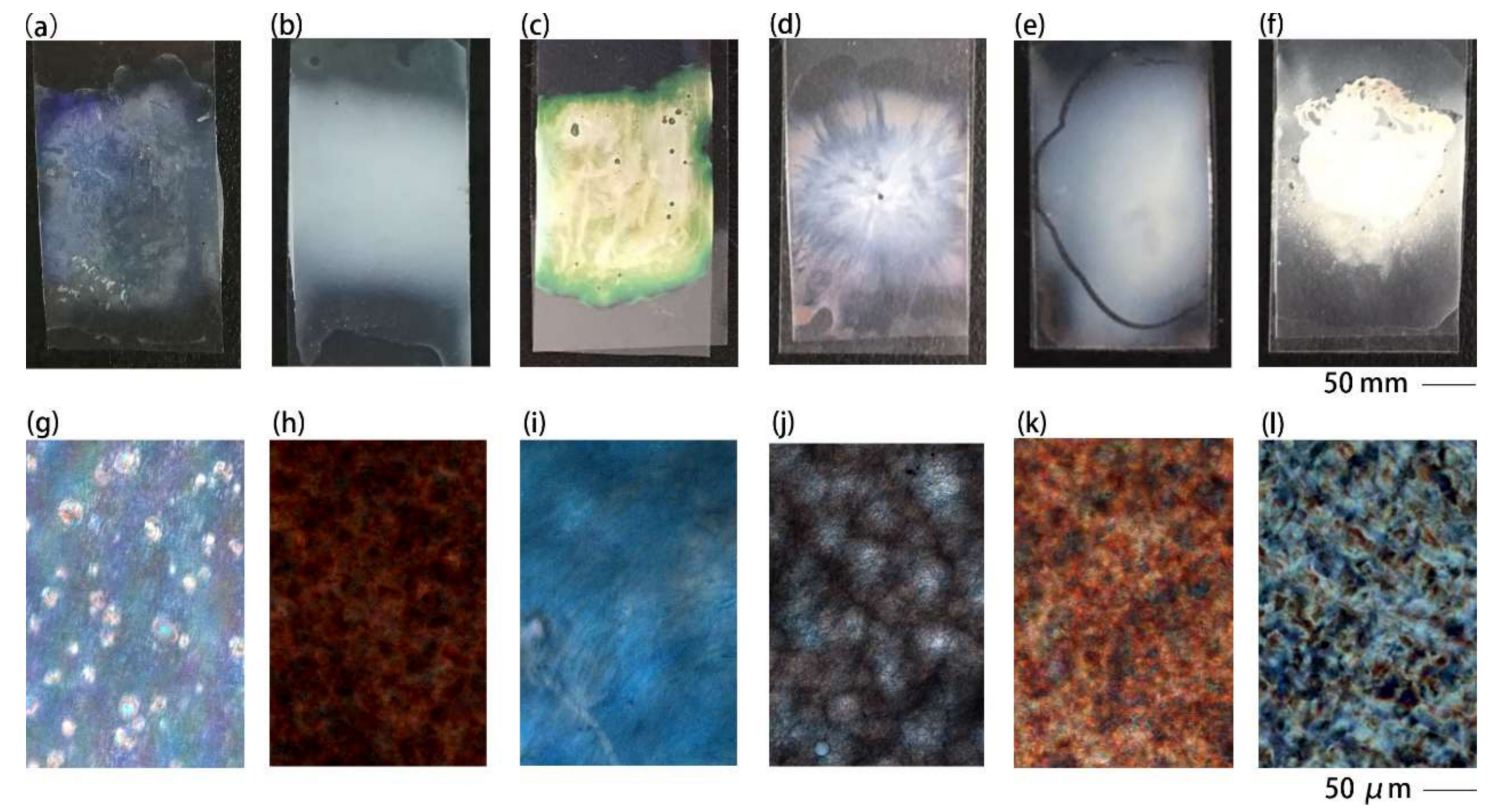
Actual pictures of the samples: (**a**) A1; (**b**) A2; (**c**) A3; (**d**) B1; (**e**) B2; (**f**) B3. Typical polarized optical microscopic images at room temperature: (**g**) A1; (**h**) A2; (**i**) A3; (**j**) B1; (**k**) B2; (**l**) B3.

As illustrated in Figure 1, Figure 1a–f shows the actual pictures of all the samples respectively. It can be found that samples A1, A2, and A3 with a polymer dispersed network by photo-polymerization were more transparent than samples B1, B2, and B3 with a polymer dispersed network by thermal-polymerization, which was also proved by UV/Vis/IR spectrum in later measurement. Moreover, samples A1, A3, B1, and B3 displayed selective reflection characteristics, suggesting that SLC1717/S811 and ChSCLCP formed planar orientation spontaneously (which was demonstrated by the oily streak like texture under POM as shown in Figure 1g,i,j,l after introducing the polymer dispersed network. However, samples A2 and B2 could not spontaneously form a planar orientation, thus a scattering state was developed during the curing process, which was unsuitable for anti-fake use. Furthermore, samples B1 and B3 obtained by the thermal-polymerization had more scattering than samples A1 and A3 prepared by photo-polymerization, probably due to the fact that the planar orientation was somehow damaged during the heating procedure.

**Figure 2:**
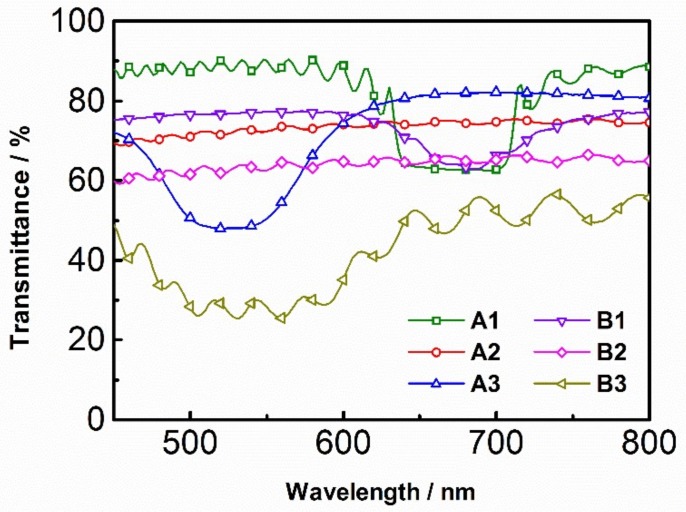
The transmission spectra of the samples.

To further investigate the light transmittance properties of the samples, UV/Vis/IR spectra were utilized and the results are shown in Figure 2. The overall transmittance and the selective reflection intensities of A1, A2, and A3 were higher than that of B1, B2, and B3, further demonstrating that the heating procedure could damage the planer orientation of ChLC, which was also in accordance with the POM results. For the samples containing the same polymer dispersed network, taking samples A1 and A3 as an example, sample A3 showed higher transmittance and stronger selective reflection than that of A1; a similar trend was also found in samples B1 and B3, indicating that ChSCLCP could perform better angular photochromic phenomenon and be more suitable for anti-fake use. However, samples A2 and B2 did not exhibit selective reflection feature; we did not take them into account for the later tests.

**Figure 3:**
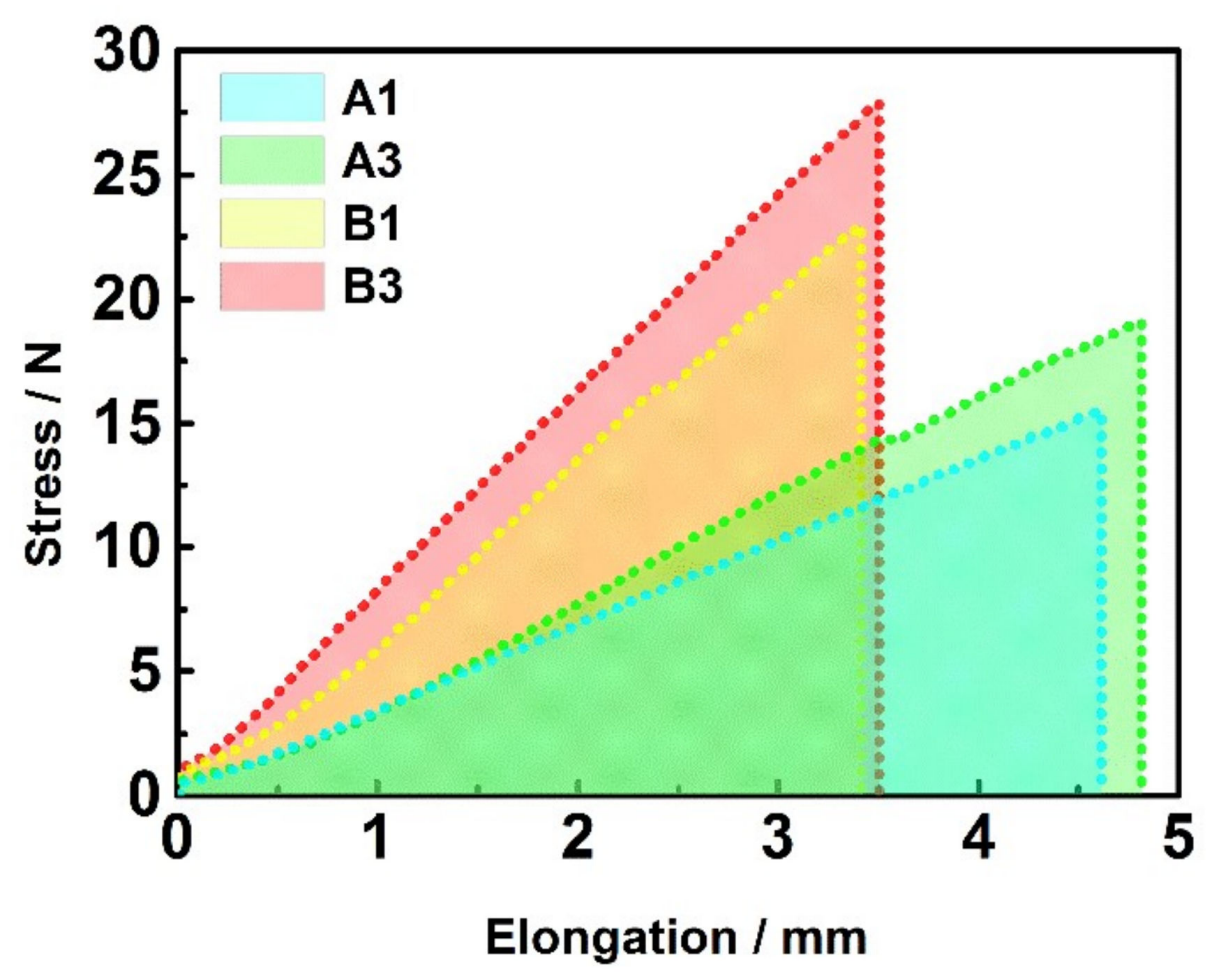
Peeling strength results of sample A1, A3, B1, and B3.

However, although the overall optical properties of the samples with a polymer dispersed network by photo-polymerization were better than that with a polymer dispersed network by thermal-polymerization, the mechanical performance of the samples showed an interesting phenomenon. As shown in Figure 3, the largest peeling strengths of samples B1 and B3 were nearly 10 N higher than that of samples A1 and A3, while the maximum elongation of the later ones showed greater improvement over the former ones, indicating that the polymer dispersed network by thermal-polymerization contributed more for peel strength improvement, and the polymer dispersed network by photo-polymerization contributed more for toughness enhancement.

**Figure 4:**
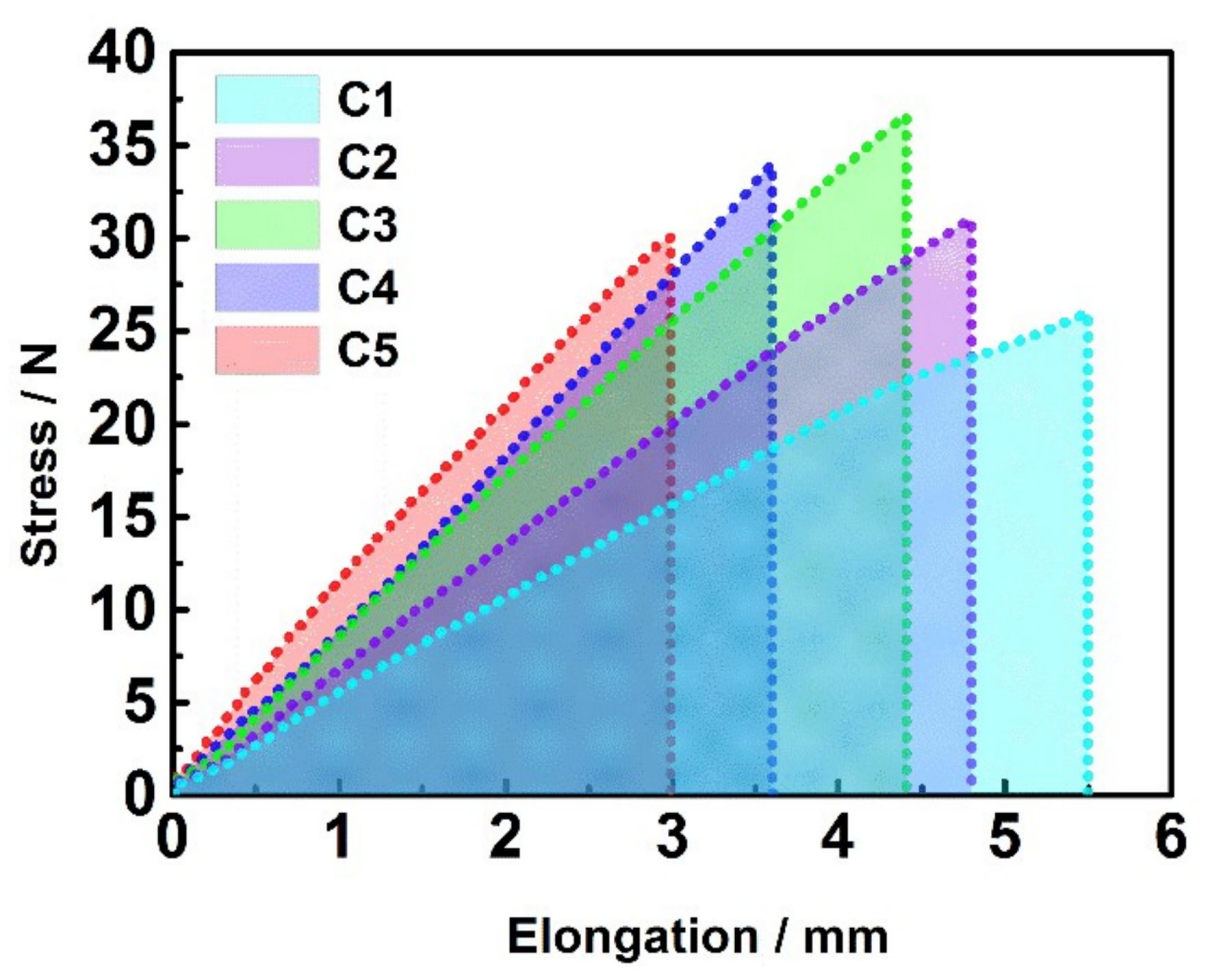
Peeling strength results of sample C1, C2, C3, C4, and C5.

From the above results, we expect that there may be some interaction between them if we introduce the two kinds of polymer dispersed networks simultaneously in one system, and an equilibrium point may exist. So a coexistent polymer dispersed network by a step polymerization of first photo-polymerization and subsequent thermal-polymerization was attempted and the effect of different ratios between the photo-polymerizable and thermal-polymerizable monomers on the mechanical properties was investigated. Figure 4 shows the mechanical property of the single and the coexistent polymer dispersed networks. As expected, the coexistent polymer dispersed network exhibited enhanced mechanical performance. When the weight ratio of the two polymerizable monomers was nearly 1:1, the film showed an overall optimal mechanical property of peeling strength and toughness.

### 3.3. Preparation of Angular Photochromic Films with a Coexistent Polymer Dispersed Network

**Figure 5:**
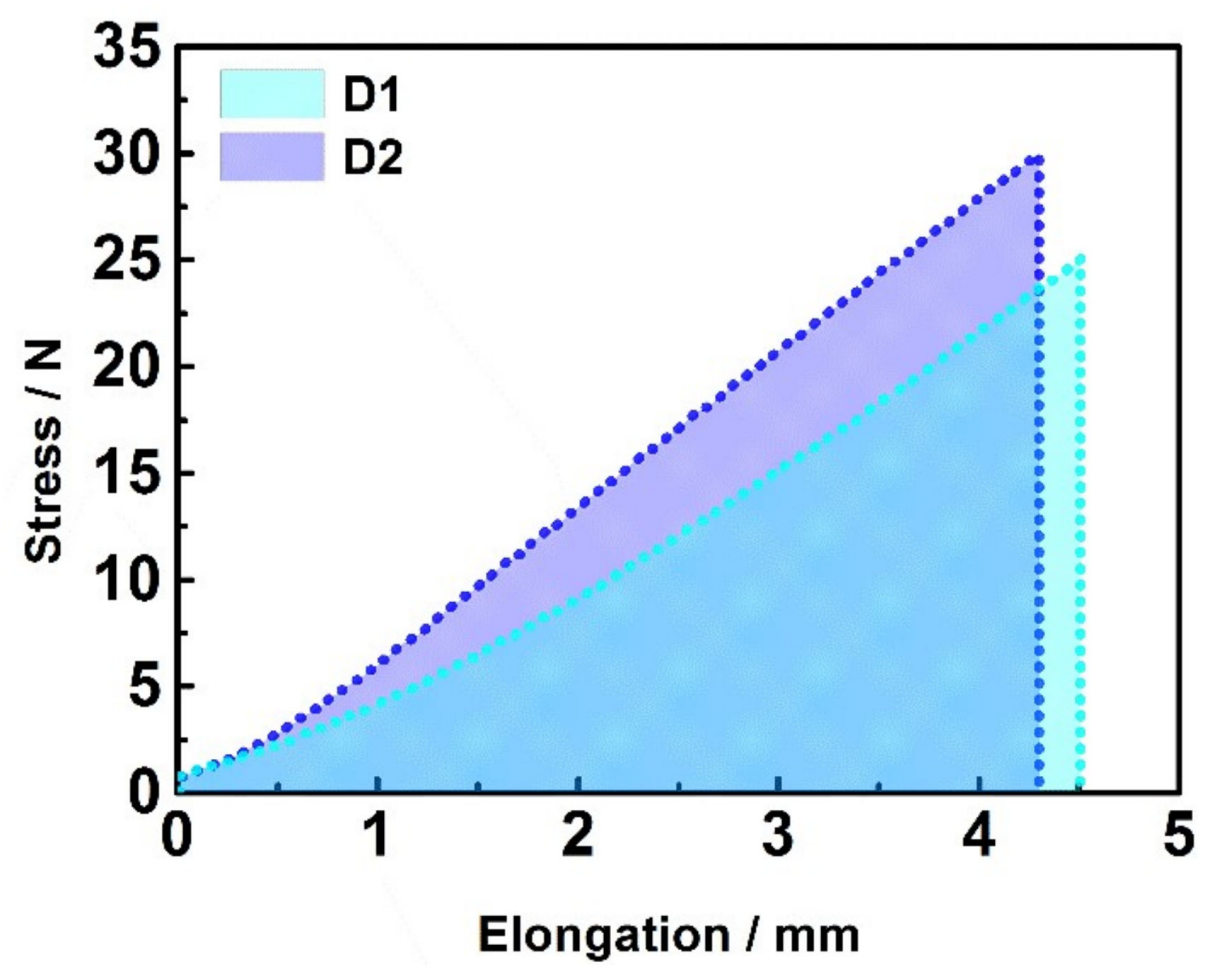
Peeling strength results of sample D1 and D2.

According to the results obtained above, when the weight ratios of photo-polymerizable and thermal-polymerizable monomers were similar, the coexistent polymer dispersed network exhibited the optimal mechanical performance, which was favorable for the anti-fake application. Thus, two kinds of angular photochromic films based on ChLC of SLC1717/S811 and 3HG2080 with a coexistent polymer dispersed network (denoted as D1 and D2, respectively) were prepared. Figure 5 shows the mechanical property of the angular photochromic films. A comparative mechanical property of peeling strength and toughness with that of the corresponding coexistent polymer dispersed network was obtained. Furthermore, comparing the mechanical properties of the two angular photochromic films, they benefited from the excellent mechanical and processable properties of the polymer materials, the film based on 3HG2080 exhibited a relatively superior performance.

**Figure 6:**
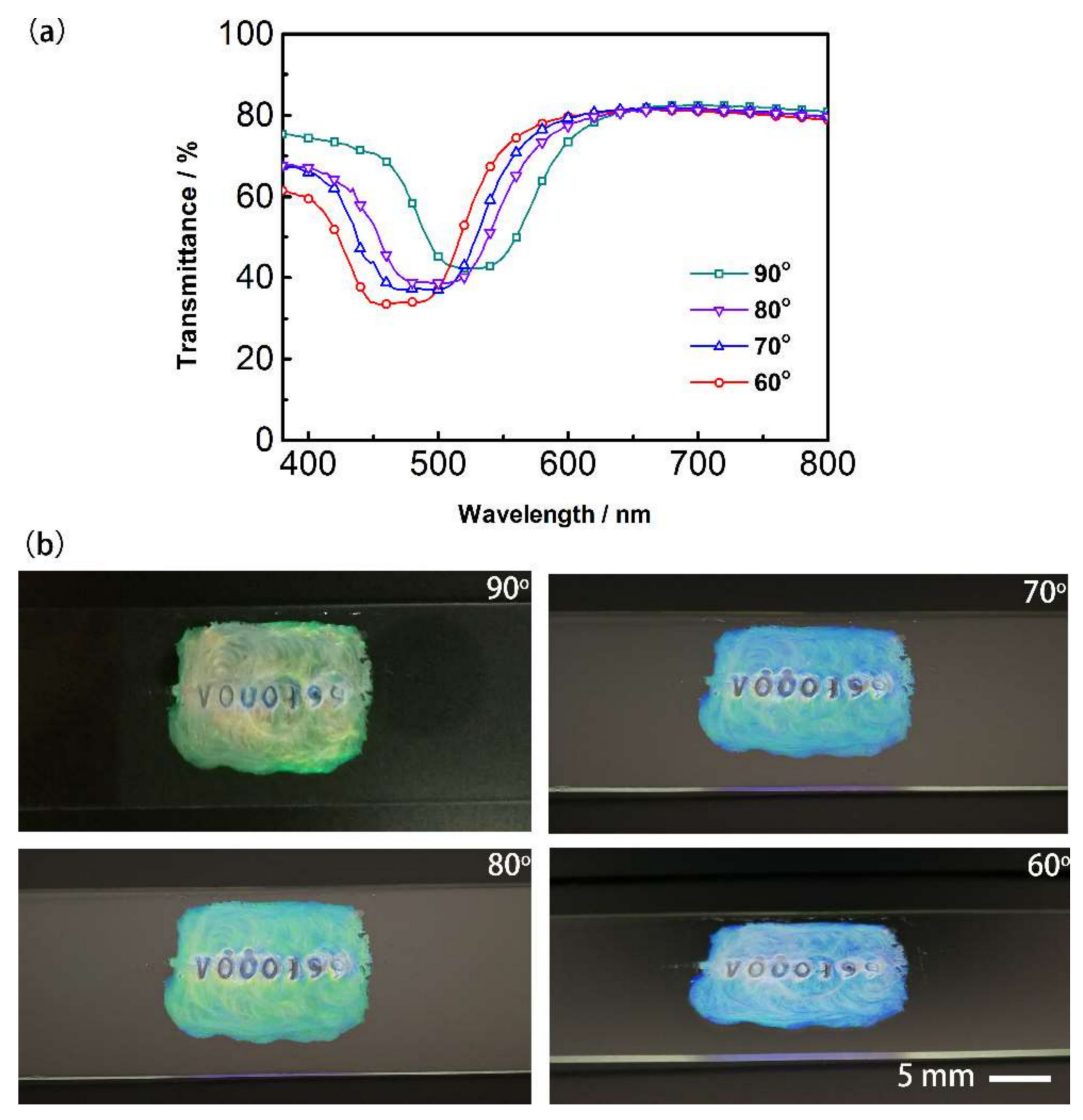
(**a**) The transmission spectra of sample D2 from different view angles; (**b**) pictures of the angular photochromic anti-fake label with different viewing angles.

Figure 6a show the typical profile of the light transmittance spectra of sample D2 in the visible region. The angular photochromic region was more than 100 nm wide with different viewing angles. An angular photochromic label with 10 mm × 10 mm was manufactured based on 3HG2080 with a coexistent polymer dispersed network. Accordingly, as shown in Figure 6b, when the viewing angle varied from 90° to 60°, significant color changes from green, cyan, blue, and purple could be observed, indicating that an angular photochromic label for anti-fake purpose had been successfully obtained.

## 4. Conclusions

In summary, employing the selective light reflection characteristic of ChLC, by introduction of a coexistent polymer dispersed network via a stepwise polymerization of first photo-polymerization and subsequently thermal-polymerization, an angular photochromic anti-fake film with enhanced mechanical and apparent angular discoloration characteristics was successfully developed. Detailed investigation found that the polymer networks developed by different methods had different effects on the mechanical properties: the polymer dispersed network by thermal-polymerization contributed more for peel strength improvement, and the polymer dispersed network by photo-polymerization contributed more for toughness enhancement. It is believed that the ChLC anti-fake film will have practical application in the negotiable instrument field such as for currencies, bank documents, and so on.

## Figures and Tables

**Table 1:** The compositions of all the samples.

ChLC	Contents	Phase Transition ^b^/°C	Selective Reflection Wavelength/nm
S1	SLC1717/S811 3/1 ^a^	Cr −15.5 Ch 83.1 Iso	668
S2	C6M/S811 3/1 ^a^	Cr 77.6 Ch 106.8 Iso	675
S3	3HG2080	g 59.0 Ch 215.5 Iso	525

^a^ Weight ratio; ^b^ According to differential scanning calorimeter (DSC) results during the cooling scan at a rate of 10 °C/min, Cr: crystallization temperature; g: glass-transition temperature; Ch: cholesteric phase; Iso: isotropic phase.

**Table 2:** The compositions of all the samples.

Sample	UA ^a^/IPDI ^b^/TTEG ^c^/SLC1717/C6M/S811/3HG2080 ^a^Weight Ratio	Polymerization Method
A1	20/0/0/60/0/20/0	Photo-polymerization
A2	20/0/0/0/60/20/0	Photo-polymerization
A3	20/0/0/0/0/0/80	Photo-polymerization
B1	0/10/10/60/0/20/0	Thermo-polymerization
B2	0/10/10/0/60/20/0	Thermo-polymerization
B3	0/10/10/0/0/0/80	Thermo-polymerization
C1	100/0/0/0/0/0/0	Photo-polymerization
C2	25/37.5/37.5/0/0/0/0	Photo- and thermo-polymerization
C3	50/25/25/0/0/0/0	Photo- and thermo-polymerization
C4	75/12.5/12.5/0/0/0/0	Photo- and thermo-polymerization
C5	0/50/50/0/0/0/0	Thermo-polymerization
D1	10/5/5/60/0/20/0	Photo- and thermo-polymerization
D2	10/5/5/0/0/0/80	Photo- and thermo-polymerization

^a^ Urethane acrylate, a photo-polymerizable monomer; ^b^ poly-isophorone di-isocyanate, a thermo-polymerizable monomer; ^c^ tetra-ethylene glycol, a thermo-polymerizable monomer.
